# *BRAF*^*V600E*^ promotes DC3/monocyte differentiation in human gene-engineered HSPCs and causes multisystem histiocytosis

**DOI:** 10.1038/s41375-023-02019-3

**Published:** 2023-09-06

**Authors:** Tommaso Sconocchia, Johannes Foßelteder, Lisa Auinger, Erdem Özkaya, Theresa Benezeder, Magdalena Lang, Christine Beham-Schmid, Gerald Hoefler, Armin Zebisch, Albert Wölfler, Heinz Sill, Peter Wolf, Herbert Strobl, Andreas Reinisch

**Affiliations:** 1 Division of Hematology, Department of Internal Medicine, Medical University of Graz, Graz, Austria; 2 Department of Dermatology, Medical University of Graz, Graz, Austria; 3 Division of Immunology, Otto Loewi Research Center for Vascular Biology, Immunology, and Inflammation, Medical University of Graz, Graz, Austria; 4 Diagnostic & Research Institute of Pathology, Medical University of Graz, Graz, Austria; 5 Division of Pharmacology, Otto Loewi Research Center for Vascular Biology, Immunology, and Inflammation, Medical University of Graz, Graz, Austria; 6 Department of Blood Group Serology and Transfusion Medicine, Medical University of Graz, Graz, Austria

**Keywords:** Haematological cancer, Immunological disorders

## To the Editor:

In the last decade, the gain-of-function *BRAF*^*V600E*^ mutation was observed to be recurrent in the histiocytic disorders Langerhans cell histiocytosis (LCH) and Erdheim-Chester disease (ECD), characterized by the infiltration of CD1a^+^CD207^+^ and CD68^+^CD1a^−^ clonal mononuclear phagocytes, respectively [1]. The *BRAF*^*V600E*^ mutation can be detected not only in terminally differentiated cells but also in hematopoietic stem and progenitor cells (HSPCs) suggesting a hematopoietic stem cell origin of these diseases [2]. Moreover, the detection of *BRAF*^*V600E*^-mutated HSPCs is associated with severe multisystem disease [3]. The constitutive activation of the MAPK pathway in LCH and ECD was shown to promote oncogene-induced senescence (OIS) with expression of anti-apoptotic proteins, cyclin-dependent kinase inhibitors (CDKI), and a senescence-associated secretory phenotype (SASP) characterized by increased expression of proinflammatory cytokines/chemokines [4–6].

Although the understanding of LCH and ECD has greatly advanced leading to better treatment and diagnosis, the difficulty in obtaining clinical material is limiting further translational progress. In addition, front-line chemotherapy and even targeted therapy of patients with multisystem disease often leads to relapse upon discontinuation of treatment, emphasizing the need for alternative treatment strategies [7]. Therefore, the development of novel pre-clinical models would help improve the molecular understanding of these diseases and identify new treatment strategies.

**Fig. 1:**
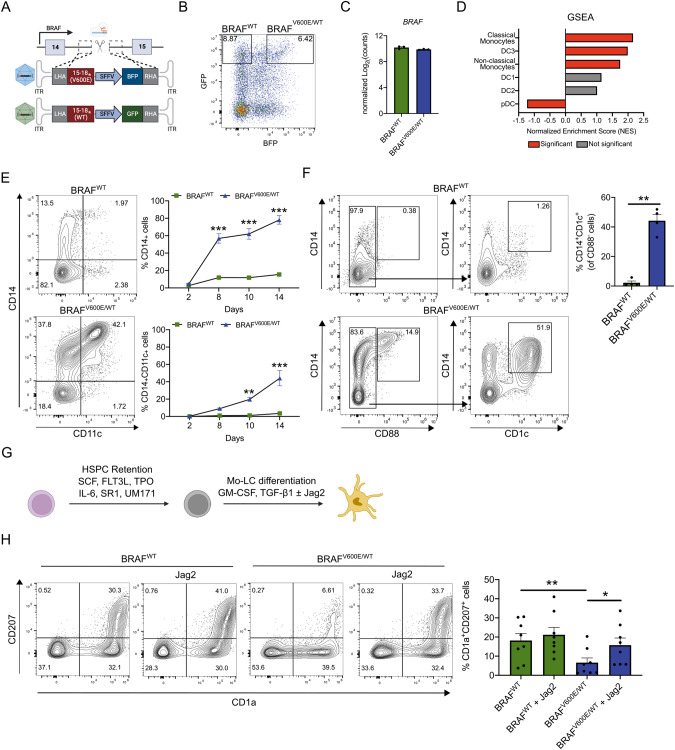
CRISPR/Cas9 gene-engineered *BRAF*^*V600E/WT*^ HSPCs spontaneously gain characteristics of monocyte/DC3-like cells and require Notch signaling to acquire LCH-like characteristics. **A** Descriptive graphic illustrating the integration by homology-directed repair of the BRAF^WT^ and BRAF^V600E^ templates in the intron 14 of the *BRAF* gene following a double-stranded break introduction by CRISPR/Cas9. The repair templates are packaged in a rAAV and include 400 bp long right and left homology arms and a fluorescent reporter protein under the control of the SFFV promoter. Asterisk (*) indicates the presence of a stop codon. **B** Representative flow cytometry plot depicting the expression levels of GFP and BFP 2 days after transfection with the RNP complex and transduction with rAAVs. Cells single positive for GFP represent *BRAF*^*WT*^ HSPCs and cells double positive for GFP and BFP represent *BRAF*^*V600E/WT*^ HSPCs. **C** Log_2_ transformed normalized expression counts for *BRAF* (*n* = 3). **D** Gene set enrichment analysis (GSEA) performed on *BRAF*^*V600E/WT*^ vs. *BRAF*^*WT*^ HSPCs. Gene sets were obtained from Villani et al. [15] and are ranked based on the normalized enrichment score (NES). Red bars represent gene sets that reached significance (*p* < 0.05) and gray bars represent gene sets that did not reach significance. **E** Representative flow cytometry plots and graphs showing the percentages of CD14^+^ and CD14^+^CD11c^+^ cells between 2 and 14 post-editing (*n* = 5). **F** Representative flow cytometry plots and graphs depicting the expression of CD88, CD14, and CD1c 10 days post-editing (*n* = 4). **G** Scheme outlying the mo-LC promoting culture conditions in the presence or absence of a Notch agonist. **H** Representative flow cytometry plots and graph showing the percentages of CD1a^+^CD207^+^ cells following a 6 day culture in the conditions described in (**G**). Data are shown as mean ± SEM. *p < 0.05, **p < 0.01, ***p < 0.001.

In this study, we developed a novel model to investigate the effects of the *BRAF*^*V600E*^ mutation in human HSPCs. We introduced a heterozygous *BRAF*^*V600E*^ mutation into human HSPCs by adapting our previously described knock-in strategy [8]. This strategy relies on the use of CRISPR/Cas9 and recombinant adeno-associated virus serotype 6 (rAAV6) as vectors for donor template delivery. Importantly, simultaneous transduction with two rAAV6s, one encoding *BRAF*^*V600E*^ and the other encoding wild-type *BRAF* (*BRAF*^*WT*^*)* allowed us to engineer heterozygous mutant cells. A green and blue fluorescent protein expression cassette (GFP and BFP) was included downstream of the BRAF^WT^ and BRAF^V600E^ cDNA respectively, to properly track and purify edited cells by FACS (Supplementary Fig. 1A and Fig. 1A). Following this strategy, heterozygously mutated *BRAF*^*V600E/WT*^ (GFP^+^BFP^+^) and *BRAF*^*WT*^ HSPCs (GFP^+^) were generated (Fig. 1B). Insertion of the mutation was confirmed by Sanger sequencing, and immunocytochemistry. Moreover, constitutive activation of the MAPK pathway was confirmed (Supplementary Fig. 1B–D).

To better understand the effects of the *BRAF*^*V600E*^ mutation on HSPCs, we performed RNA-sequencing (RNAseq) (Supplementary Fig. 2A) and identified 1604 differentially expressed genes (DEGs; 891 up-regulated and 713 down-regulated; cut-off value of ±1 log_2_ fold change) (Supplementary Fig. 2B). Importantly, RNAseq confirmed unaltered *BRAF* gene expression with our approach (Fig. 1C), representing an improvement from lentiviral-based overexpression systems, where heterologous promoters drive gene expression often from multiple viral integration sites [9].

Myeloid promoting transcription factors were up-regulated in *BRAF*^*V600E/WT *^HSPCs, whereas genes involved in HSC maintenance and erythroid differentiation were down-regulated (Supplementary Fig. 2C).

Gene set enrichment analysis (GSEA) revealed a significant enrichment of genes corresponding to CD14^+^ classical monocytes, DC3s, and CD16^+^ non-classical monocytes (Fig. 1D and Supplementary Fig. 2D).

To investigate the effects of the mutation on HSPC function, we performed colony-forming unit (CFU) assays and myeloid promoting liquid cultures. In the CFU assay, the *BRAF*^*V600E/WT *^HSPCs produced less colonies with a skewing toward monocyte/macrophage (CFU-M) colonies at the expense of erythroid (BFU-E) colonies (Supplementary Fig. 3A, B). Myeloid promoting liquid cultures (SCF, FLT3L, TPO, IL-3, GM-CSF, and G-CSF) revealed similar results with the *BRAF*^*V600E/WT *^HSPCs that proliferated less but showed increased myeloid differentiation with higher expression of monocyte/macrophage markers (Supplementary Fig. 3C–E).

We next evaluated whether the mutation itself drives myeloid differentiation, in the absence of strong myeloid-differentiation promoting cytokines (IL3, GM-CSF, G-CSF). Therefore, FACS-purified modified HSPCs were cultured in HSPC retention conditions (SCF, FLT3L, TPO, IL-6, SR1, UM171). Strikingly, under these conditions, *BRAF*^*V600E/WT *^HSPCs were characterized by a strong proliferative advantage over their WT counterparts, quickly lost CD34 expression, and gained expression of CD11b, CD14, and CD11c (Fig. 1E and Supplementary Fig. 4A–C). We identified a small percentage of cells co-expressing the monocyte makers CD16 and CD88, while most CD14^+^ cells lacked CD88 and co-expressed the dendritic cell maker CD1c, suggesting a DC3 differentiation bias induced by *BRAF*^*V600E*^ (Fig. 1F and Supplementary Fig. 4D).

Numerous *BRAF*^*V600E/WT*^ cells also morphologically appeared as large foamy cells expressing histiocyte markers CD68, CD163, and S100 (Supplementary Fig. 4E, F). In summary, our data suggest that the *BRAF*^*V600E*^ mutation induces cell differentiation to a heterogenous population enriched for DC3-like cells, monocytes, and foamy macrophages.

We further extended our characterization by including LCH markers. Interestingly, *BRAF*^*V600E/WT*^ cells did not gain CD1a and CD207 expression neither under HSPC retention conditions nor under established serum-free LC promoting conditions [10] (TGF-β1, GM-CSF, SCF, FLT3L, and TNFα) (Supplementary Fig. 5A–D) suggesting that *BRAF*^*V600E/WT *^HSPCs either lack LC potential or critical (co-)signals for LC differentiation. Because the *BRAF*^*V600E*^ mutation causes HSPCs to rapidly gain characteristics of DC3/monocytes, and Notch signaling was previously described to be required for their development into CD1a^+^CD207^+^LC-like cells [2, 11, 12], we tested whether this could be the missing stimulus. Under monocyte-derived LC (mo-LC) promoting conditions (GM-CSF, TGF-β1, and Notch ligand), the presence of the Notch agonist Jagged-2 (Jag2) resulted in a significant increase in the percentages of CD1a^+^CD207^+^ cells in the *BRAF*^*V600E/WT*^ condition, confirming that Notch ligation strongly enhances their differentiation into LC-like cells (Fig. 1G, H).

To determine whether our model could also recapitulate histiocytic disorders in vivo, we intrafemorally transplanted HSPCs engineered with *BRAF*^*WT*^ or *BRAF*^*V600E*^ into immune-compromised mice (Supplementary Fig. 6A). The presence of co-expressed GFP and BFP allowed for precise tracking of *BRAF*^*WT*^ and *BRAF*^*V600E*^-edited cells, respectively. Both groups showed comparable levels of total human engraftment (Supplementary Fig. 6B), however *BRAF*^*V600E*^ cells (BFP^+^) significantly increased overtime whereas the *BRAF*^*WT*^ cells (GFP^+^) decreased in the engrafted bone marrow (BM) (Supplementary Fig. 6C). Interestingly, the *BRAF*^*V600E*^-reconstituted mice showed signs of illness and had to be sacrificed before the 20 week endpoint (Supplementary Fig. 6D). Analysis of their organs revealed a notable but not significant spleen enlargement (Supplementary Fig. 6E).

Analysis of human hematopoietic cells in the BM (Supplementary Fig. 6F–J) and spleen (Supplementary Fig. 6K–O), revealed a strong myeloid bias (hCD33^+^) in the *BRAF*^*V600E*^-reconstituted mice with very low percentages of B-lymphoid (hCD19^+^) cells, similar to other comparable humanized mouse models [4, 5, 9]. Interestingly, a myeloid bias was also detected in the unedited (WT) fraction in mice containing *BRAF*^*V600E*^ cells, however was not seen in mice with *BRAF*^*WT*^ cells (Supplementary Fig. 6I, J, N, O). Histopathologic analysis of the spleen and liver confirmed the infiltration of human mononuclear phagocytes expressing CD68, S100, and CD207, characteristic for ECD and LCH (Supplementary Fig. 7A–C). Overall, our data confirm that this humanized mouse model can mimic a lethal mixed multisystem histiocytosis.

**Fig. 2:**
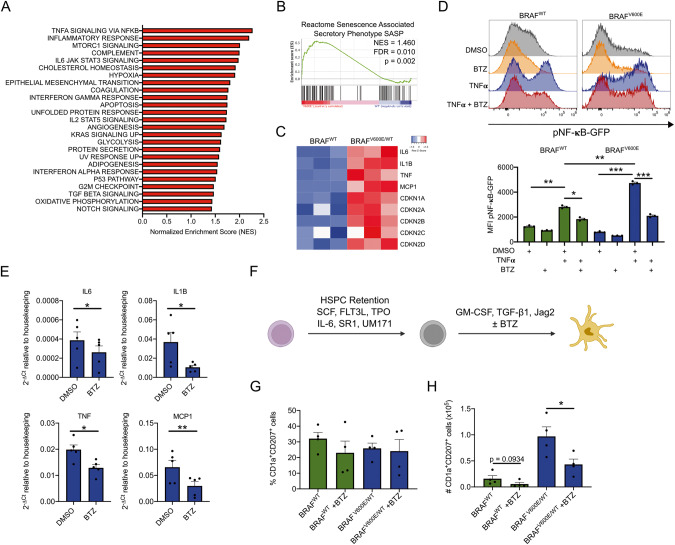
Bortezomib treatment decreases proinflammatory cytokine gene expression and inhibits CD1a^+^CD207^+^ LCH cells. **A** GSEA performed on *BRAF*^*V600E/WT*^ vs. *BRAF*^*WT*^ HSPCs for hallmark pathways. Pathways are ranked based on the normalized enrichment score (NES). **B** Enrichment plot for differentially expressed genes included in the Reactome Senescence-Associated Secretory Phenotype SASP between *BRAF*^*V600E/WT*^ and *BRAF*^*WT*^ HSPCs. **C** Heat map depicting gene expression of CDKIs and SASP cytokines (*n* = 3). **D** Graphs depicting histograms and geometric mean fluorescent intensity (MFI) of U937 *BRAF*^*V600E*^-BFP-pNF-κB-GFP reporter cells and BFP negative U937-pNF-κB-GFP cells (*BRAF*^*WT*^) treated with or without 10 ng/ml TNFα and 10 nM BTZ overnight (*n* = 3). **E** Graphs depicting the gene expression of SASP cytokines in *BRAF*^*V600E/WT*^ HSPCs following a 24 h treatment with 5 nM BTZ (*n* = 4). GAPDH was used as housekeeping gene. **F** Scheme outlining the mo-LC promoting culture conditions in the presence or absence of 5 nM BTZ of *BRAF*^*WT*^ and *BRAF*^*V600E/WT*^ HSPCs. After an initial 6 day expansion in HSPC retention medium, 4 × 10^4^ cells were cultured in the described mo-LC promoting conditions. **G** Graphs showing percentages and (**H**) absolute numbers of CD1a^+^CD207^+^ cells after 6 days of culture with GM-CSF/TGF-β1/Jag2 in the presence or absence of 5 nM BTZ (*n* = 4). DMSO was used as a vehicle control for (**D**, **E**, **G**, **H**). Data are shown as mean ± SEM. *p < 0.05, **p < 0.01, ***p < 0.001.

Proinflammatory cytokines, secreted by histiocytes and other immune-components within histiocytic lesions create a damaging microenvironment favoring the persistence of histiocytes [13]. Targeting this microenvironment could provide a therapeutic benefit for histiocytosis patients. Therefore, we utilized our model to identify enriched proinflammatory pathways. The “TNFα signaling via NF-κB pathway” was identified as the top enriched pathway (Fig. 2A) and additionally a strong enrichment for SASP genes could be detected (Fig. 2B, C). Increased expression of SASP cytokines was confirmed by qPCR and expression of CDKN2A (p16^INK4A^) was confirmed at the protein level (Supplementary Fig. 8A, B). To better evaluate the impact of *BRAF*^*V600E/WT*^ on canonical NF-κB signaling, we employed an NF-κB reporter system in the monocytic U937 cell line, engineered to carry *BRAF*^*V600E*^. Upon TNFα stimulation, *BRAF*^*V600E *^U937 showed increased NF-κB activity compared to *BRAF*^*WT*^ cells. Since TNFα was recently shown to be a key determinant of SASP in the LCH microenvironment, and its inhibition was suggested to produce a therapeutic benefit [5], we attempted to block NF-κB signaling by utilizing the clinically approved proteasome inhibitor bortezomib (BTZ), previously described to inhibit NF-κB [14]. Strikingly, BTZ was able to inhibit TNFα-induced NF-κB activation in *BRAF*^*V600E *^U937 (Fig. 2D).

Treatment of *BRAF*^*V600E/WT *^HSPCs with BTZ at a previously defined concentration of 5 nM (Supplementary Fig. 8C) was able to substantially reduce the gene expression levels of several SASP cytokines (Fig. 2E) with only minor influence on the surface expression of the myeloid markers (Supplementary Fig. 8D, E). We next sought to investigate whether BTZ could interfere with LC differentiation of mutated HSPCs. Interestingly, under mo-LC promoting conditions, BTZ significantly decreased the total number of differentiated CD1a^+^CD207^+^ cells without affecting their percentages (Fig. 2F–H). Altogether, this confirms NF-κB signaling as a key player in *BRAF*^*V600E*^-driven pathogenesis and suggests NF-κB inhibition by BTZ can be harnessed as a novel therapeutic strategy to dampen the expression of SASP cytokines and to limit the proliferation of LCH cells.

In conclusion, our novel model faithfully reproduced the effects of *BRAF*^*V600E*^-driven constitutive RAS-RAF-MEK-ERK pathway with a selective mutation-driven differentiation bias of HSPCs toward the DC3/monocyte lineage, strong enrichment in inflammatory pathways, and increased expression of SASP genes. We additionally showed that Notch signaling is necessary to promote the differentiation of *BRAF*^*V600E/WT *^HSPCs into CD1a^+^CD207^+^LCH cells. In vivo, mice transplanted with *BRAF*^*V600E *^HSPCs developed a lethal multisystem histiocytosis and this humanized mouse model can be utilized for investigating pre-clinically novel therapeutic strategies. Finally, we could demonstrate that the clinically approved drug bortezomib, by reducing the expression of SASP cytokines and inhibiting CD1a^+^CD207^+^LCH cell proliferation, is an interesting drug to be repurposed for the treatment of LCH and ECD.

### Supplementary information

Sconocchia et al Supplemental Figures and Material

## Data Availability

Data obtained from the RNA-sequencing analysis were publicly deposited and are available at GEO (GSE230040).
