# Supplementary material for: BRAFV600E promotes DC3/monocyte differentiation in human gene-engineered HSPCs and causes multisystem histiocytosis
Source: Leukemia. 2023 Sep 6;37(11):2292–6. doi: 10.1038/s41375-023-02019-3 (PMC10624620; doi:10.1038/s41375-023-02019-3)
Supplement: Supplementary file 1 — Sconocchia et al Supplemental Figures and Material [file 41375_2023_2019_MOESM1_ESM.docx]

**Supplementary Figures**

**
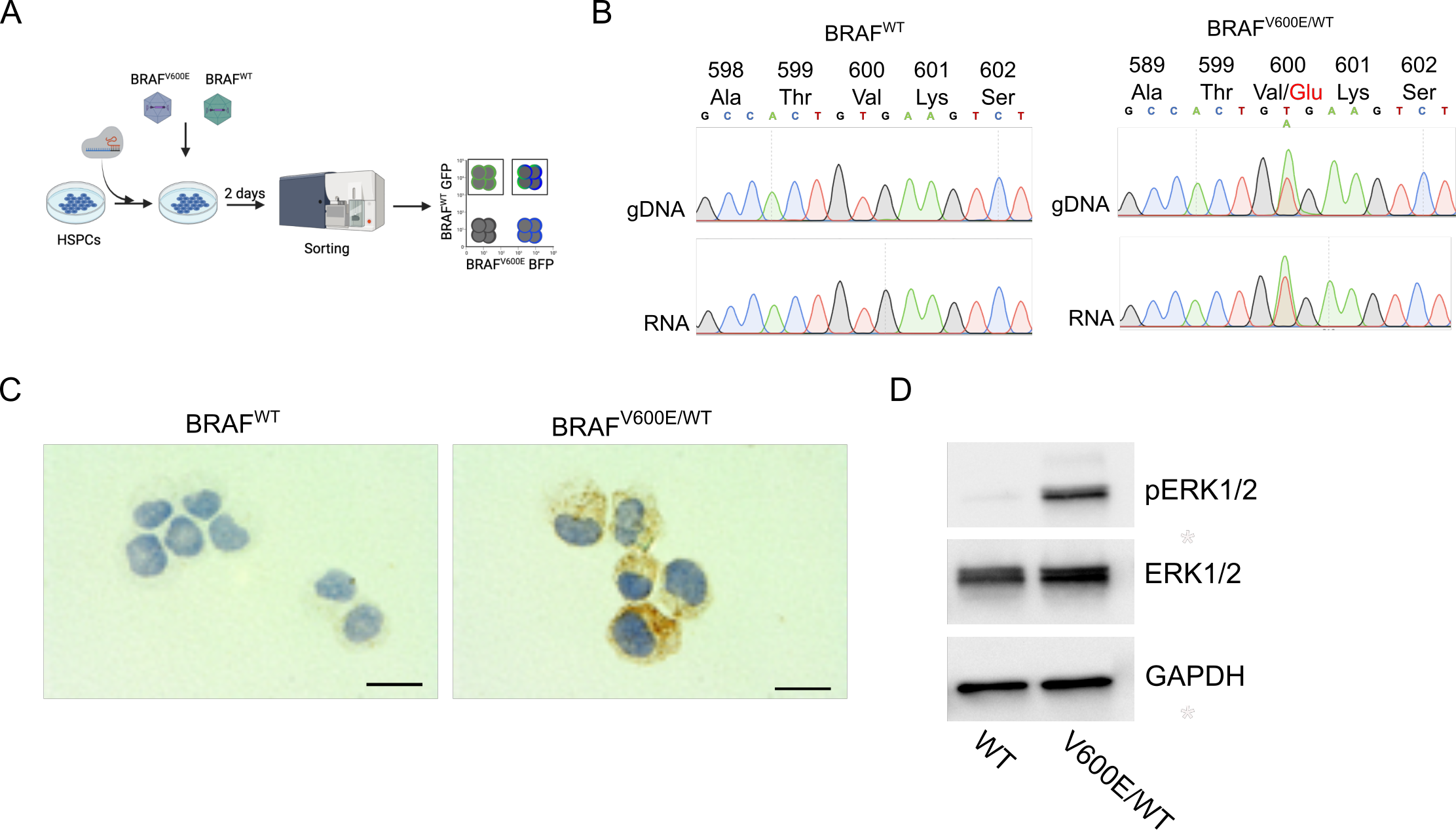
**

**Supplementary Figure 1. Engineering *BRAF^V600E/WT^* HSPCs by CRISPR/Cas9 genome editing and rAAV6 transduction.** (A) Scheme outlying the experimental design used for the generation and isolation of *BRAF^WT^* and *BRAF^V600E/WT^* HSPCs. (B) Sanger sequencing of genomic DNA (gDNA) and reversely-transcribed RNA confirming the insertion of the heterozygous *BRAF^V600E^* mutation in sort-purified *BRAF^V600E/WT^* HSPCs. (C) Confirmation of the expression of the BRAF^V600E^ protein in sort-purified *BRAF^V600E/WT^* HSPCs by immunohistochemical analysis. *BRAF^WT^* HSPCs were used as a negative control. Scale bar = 20 µm. (D) Representative western blot analysis performed on protein isolated from sort-purified *BRAF^WT^* and *BRAF^V600E/WT^* HSPCs showing the expression of ERK1/2 and phosphorylated ERK1/2. GAPDH was used as a loading control.

**
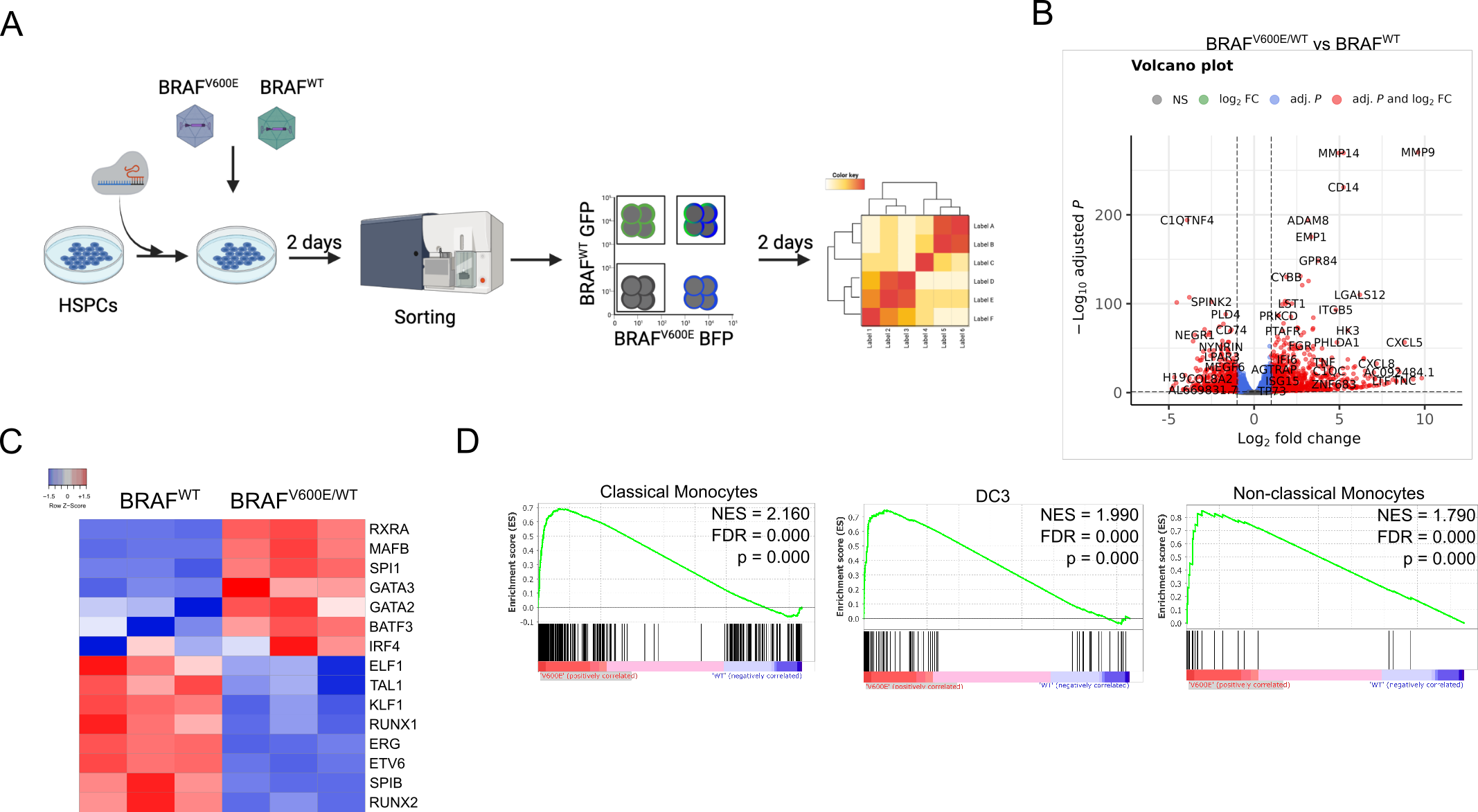
**

**Supplementary Figure 2. Gene expression analysis of *BRAF^V600E/WT^* HSPCs.** (A) Summary scheme depicting the experimental layout for the RNAseq analysis. Cells were sorted 2 days post-editing and subsequently cultured for another 2 days in HSPC retention medium before RNA extraction. (B) Volcano plot showing up- and down-regulated genes between *BRAF^V600E/WT^* vs. *BRAF^WT^* HSPCs (n=3). Blue dots represent statistically significant differentially expressed genes. Red dots represent genes with a log_2_ fold change above 1 or below 1. (C) Heat map depicting gene expression of genes involved in hematopoiesis (n=3). (D) Representative enrichment plots from selected gene sets from the GSEA.

**
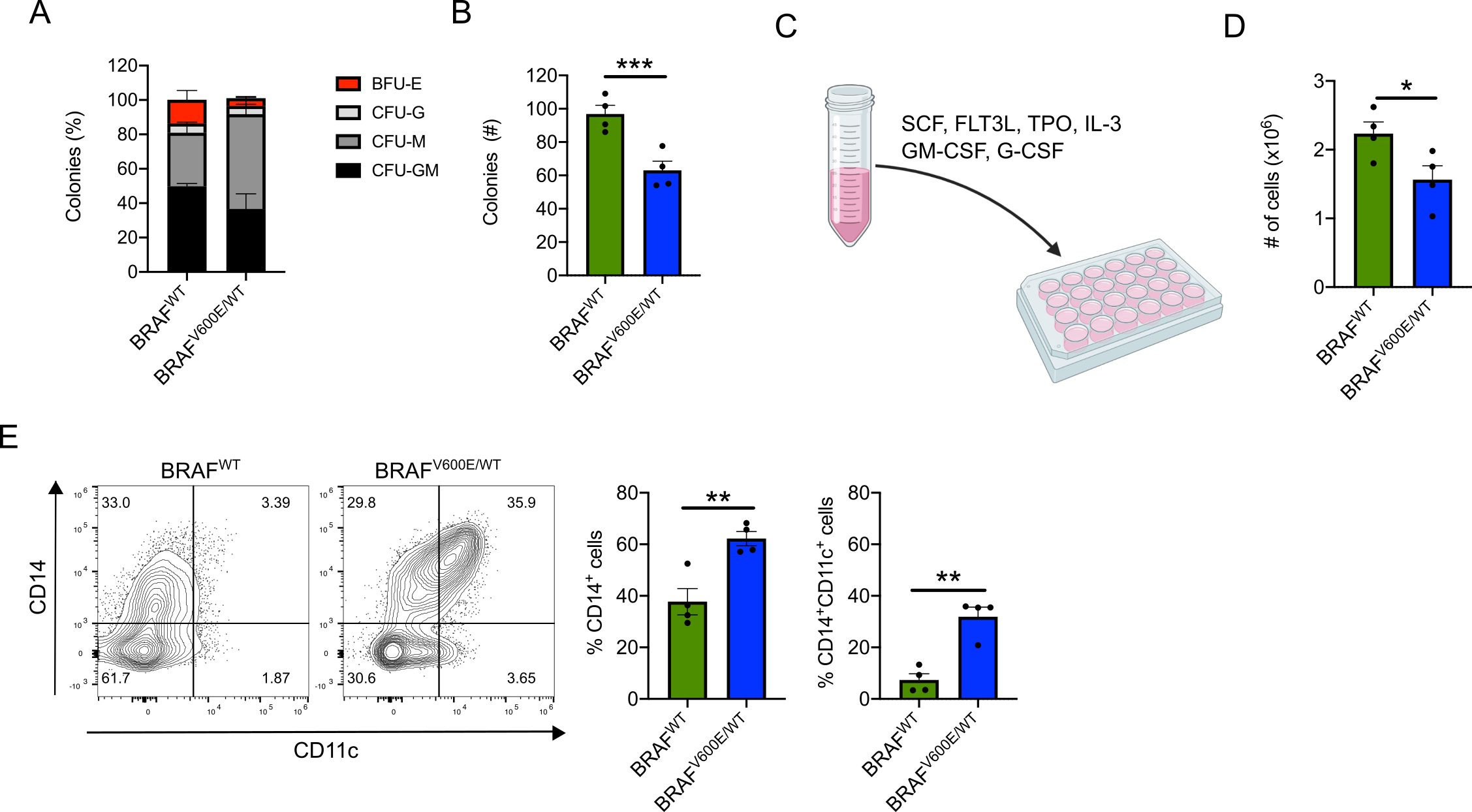
**

**Supplementary Figure 3. *BRAF^V600E^* mutation skews the differentiation of HSPCs towards the monocyte/macrophage lineage.** (A) Bar graph depicting percentages of each colony type and (B) the total number of colonies following a 14 day colony-forming unit assay performed in semisolid medium (n=4). Each dot represents a separate donor and is the mean of the 3 technical replicates. (C) Scheme outlying the myeloid promoting culture conditions. (D) Bar graph depicting the total number of cells after 6 days of culture in myeloid promoting conditions (n=4). (E) Representative flow cytometry plots and bar graphs depicting the percentage of cells expressing CD14 and/or CD11c after 6 days of culture in myeloid promoting conditions (n=4). Data are shown as mean ± SEM. *P<0.05, **P<0.01, ***P<0.001.

**
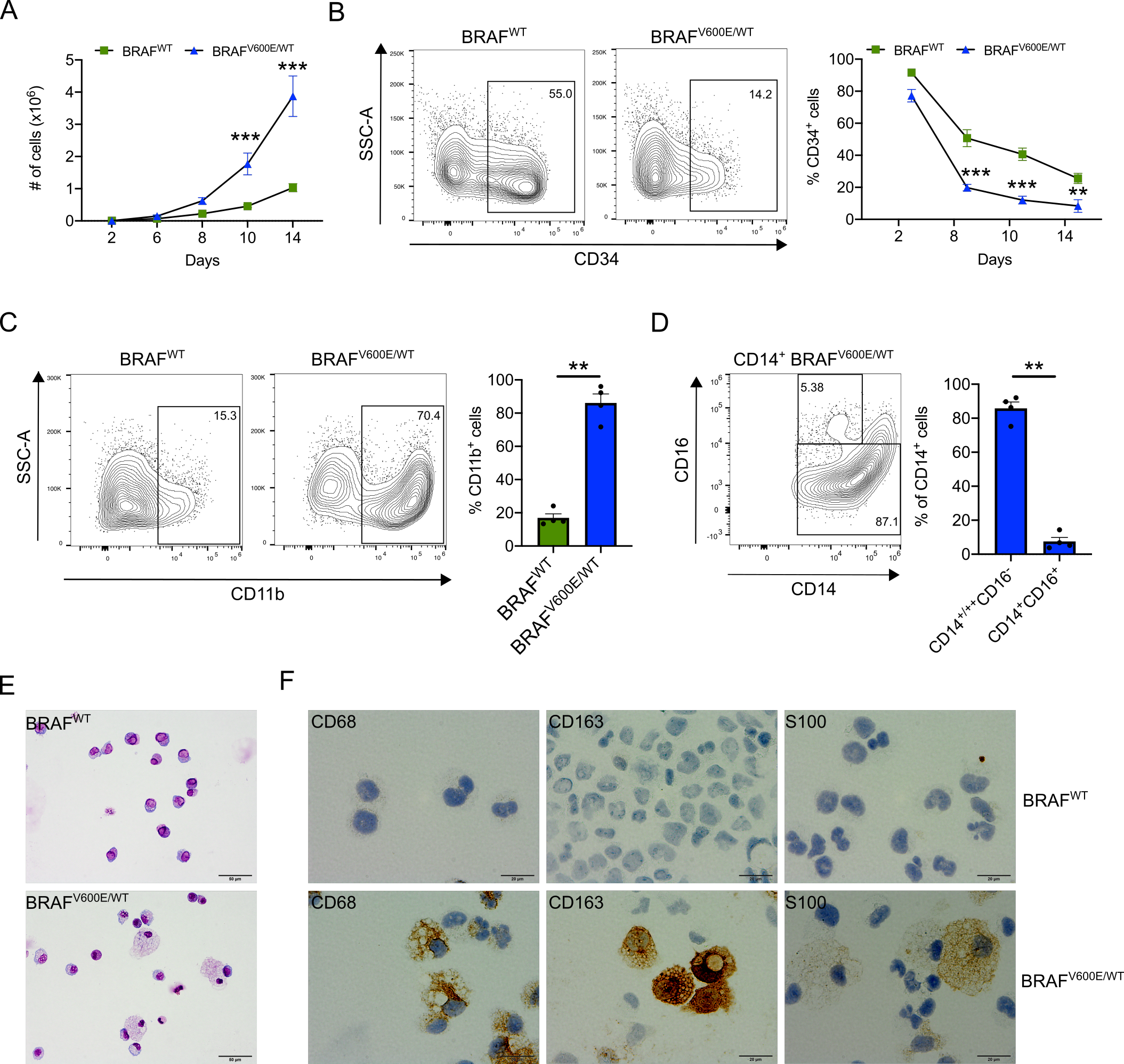
**

**Supplementary Figure 4. *BRAF^V600E/WT^* HSPCs spontaneously gain characteristics of mononuclear phagocytes.** (A) Proliferation of *BRAF^WT^* and *BRAF^V600E/WT^* HSPCs cultured in HSPC retention medium for 14 days (n=5). (B) Representative flow cytometry plots and graph showing the percentages of CD34^+^ cells between 2 and 14 days post-editing (n=5). (C) Representative flow cytometry plots and graph showing the percentages of CD11b^+^ cells 8 days post-editing (n=4). (D) Representative flow cytometry plots and graph depicting the expression of CD14 and CD16 in pre-gated CD14^+^ *BRAF^V600E/WT^* cells 10d post-editing (n=4). (E) Pappenheim staining and (F) CD68, CD163, and S100 expression of sort-purified *BRAF^WT^* and *BRAF^V600E/WT^* HSPCs after 8 days of culture in HSPC retention medium. Scale bar = 20 µm. Data are shown as mean ± SEM. **P<0.01, ***P<0.001.

**
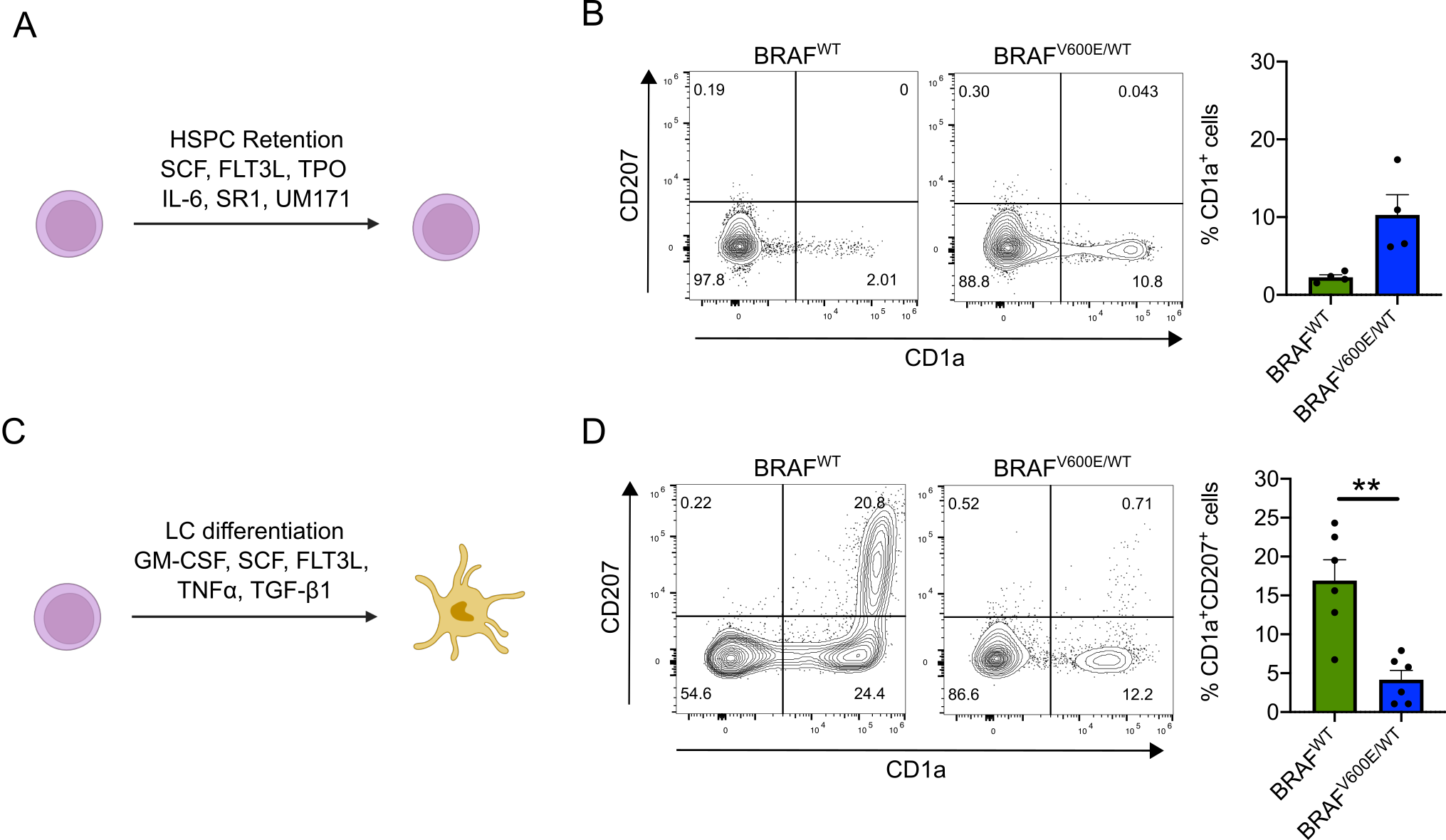
**

**Supplementary Figure 5.** ***BRAF^V600E^* and TGF-β1 are not sufficient for promoting LCH-like cells.** (A) Scheme outlying the HSPC retention culture conditions. (B) Representative flow cytometry plots and graph showing the percentages of CD1a^+^ cells (n=4) following an 8 day culture in the conditions described in A. (C) Scheme outlying the LC promoting culture conditions. (D) Representative flow cytometry plots and graph showing the percentages of CD1a^+^CD207^+^ cells (n=6) following a 7 day culture in the conditions described in C. **P<0.01.

**
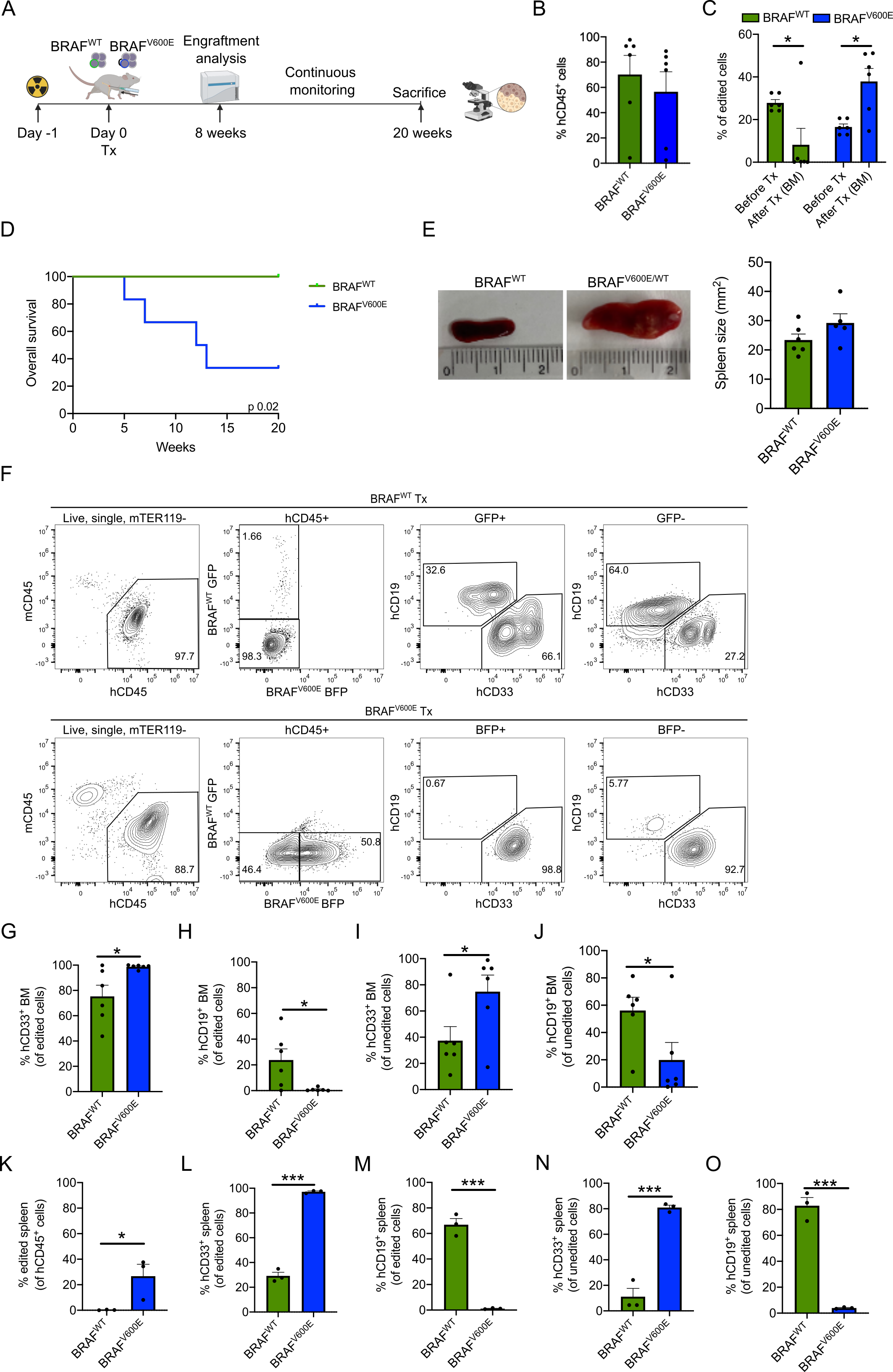
**

**Supplementary Figure 6. Human gene-engineered *BRAF^V600E^* HSPCs robustly engraft in immune deficient mice.** (A) Experimental design for the transplantation of gene-engineered HSPCs. *BRAF^WT^* or *BRAF^V600E^* HSPCs were intrafemorally transplanted into sub-lethally irradiated NSGW41 mice. BM aspirations were performed 8 weeks post-transplantation to assess engraftment levels. (B) Graph depicting the engraftment of human cells at the time of the first BM aspiration (≤ 8 weeks). Engraftment was considered by measuring the percentage of hCD45^+^ cells (n=6). (C) Graph depicting the percentage of edited cells within the total CD34^+^ HSPCs at the time of transplantation and the percentage of edited cells in the BM at the time of the first engraftment analysis measured as percentage of edited cells from total hCD45^+^ cells (n=6). (D) Kaplan-Meier curves depicting the overall survival of the mice transplanted with either *BRAF^WT^* or *BRAF^V600E^* HSPCs (n=6 mice per group). (E) Representative images of spleens collected from transplanted mice (left) and bar graph depicting the area (mm^2^) of the spleens (n=6 and 5). (F) Representative flow cytometry plots depicting the gating strategy utilized for the analyses of the mice. Percentages of (G) human myeloid (hCD33^+^), and (H) lymphoid cells (hCD19^+^) within edited cells (GFP^+^ or BFP^+^) in the BM (n=6). Percentages of (I) human myeloid (hCD33^+^), and (J) lymphoid cells (hCD19^+^) within unedited cells (GFP or BFP negative) in the BM (n=6). (K) Percentages of edited cells within total hCD45^+^ cells in the spleen (n=3). Percentages of (L) human myeloid (hCD33^+^), and (M) lymphoid cells (hCD19^+^) within edited cells (GFP^+^ or BFP^+^) in the spleen (n=3). Percentages of (N) human myeloid (hCD33^+^), and (O) lymphoid cells (hCD19^+^) within unedited cells (GFP or BFP negative) in the spleen (n=3). Data are shown as mean ± SEM. *P<0.05, ***P<0.001.

**
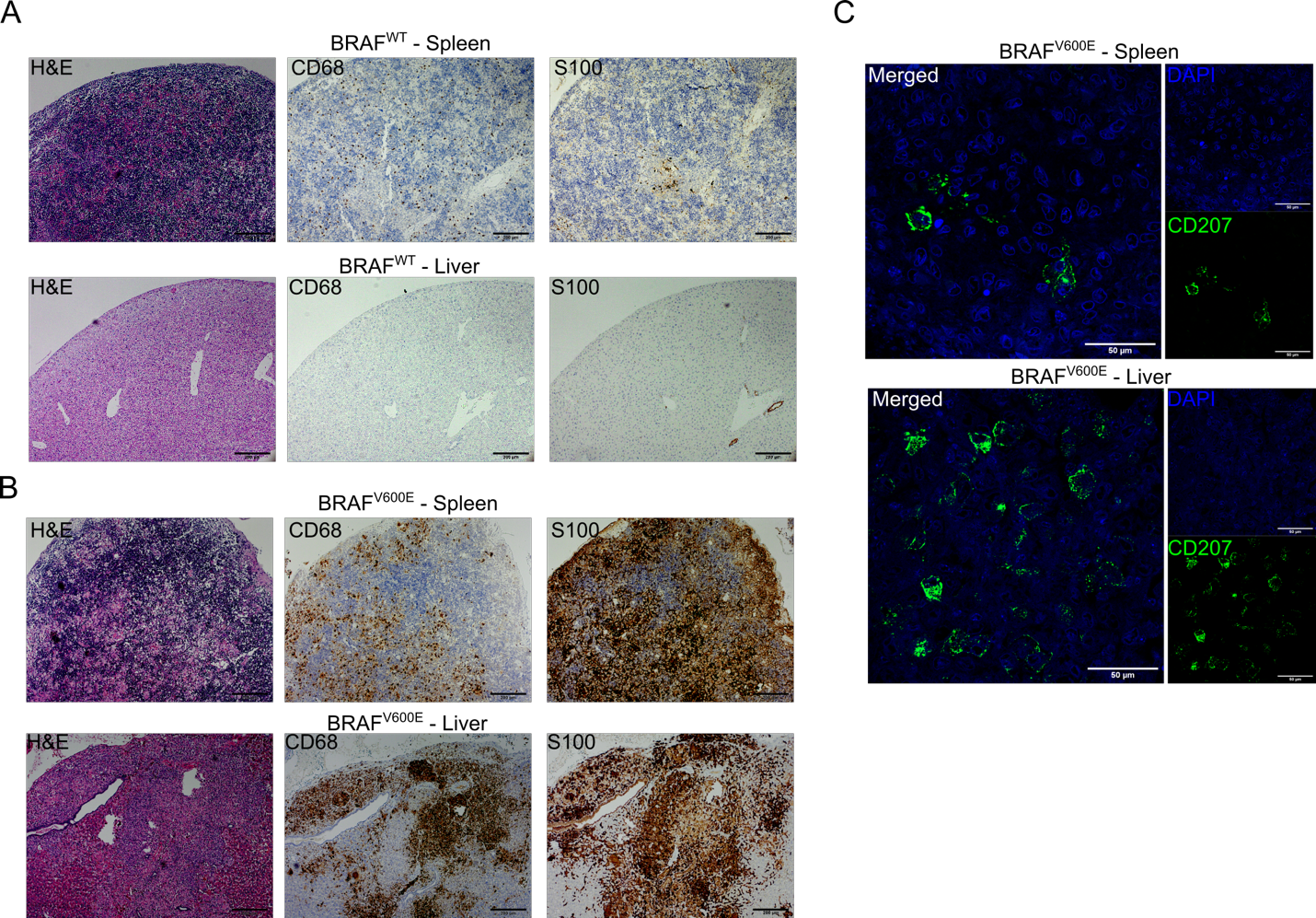
**

**Supplementary Figure 7. Xenotransplantation of *BRAF^V600E^* HSPCs promotes a mixed-histiocytic disorder in immune deficient mice.** (A) Representative images of H&E, CD68, and S100 stained spleen and liver sections of *BRAF^WT^* transplanted mice. (B) Representative images of H&E, CD68, and S100 stained spleen and liver sections of *BRAF^V600E^* transplanted mice. Scale bars = 200 µM. (C) Representative images of CD207 stained spleen and liver sections of *BRAF^V600E^* transplanted mice. DAPI was used as a nuclear counterstain. Scale bar = 50 µM.

**
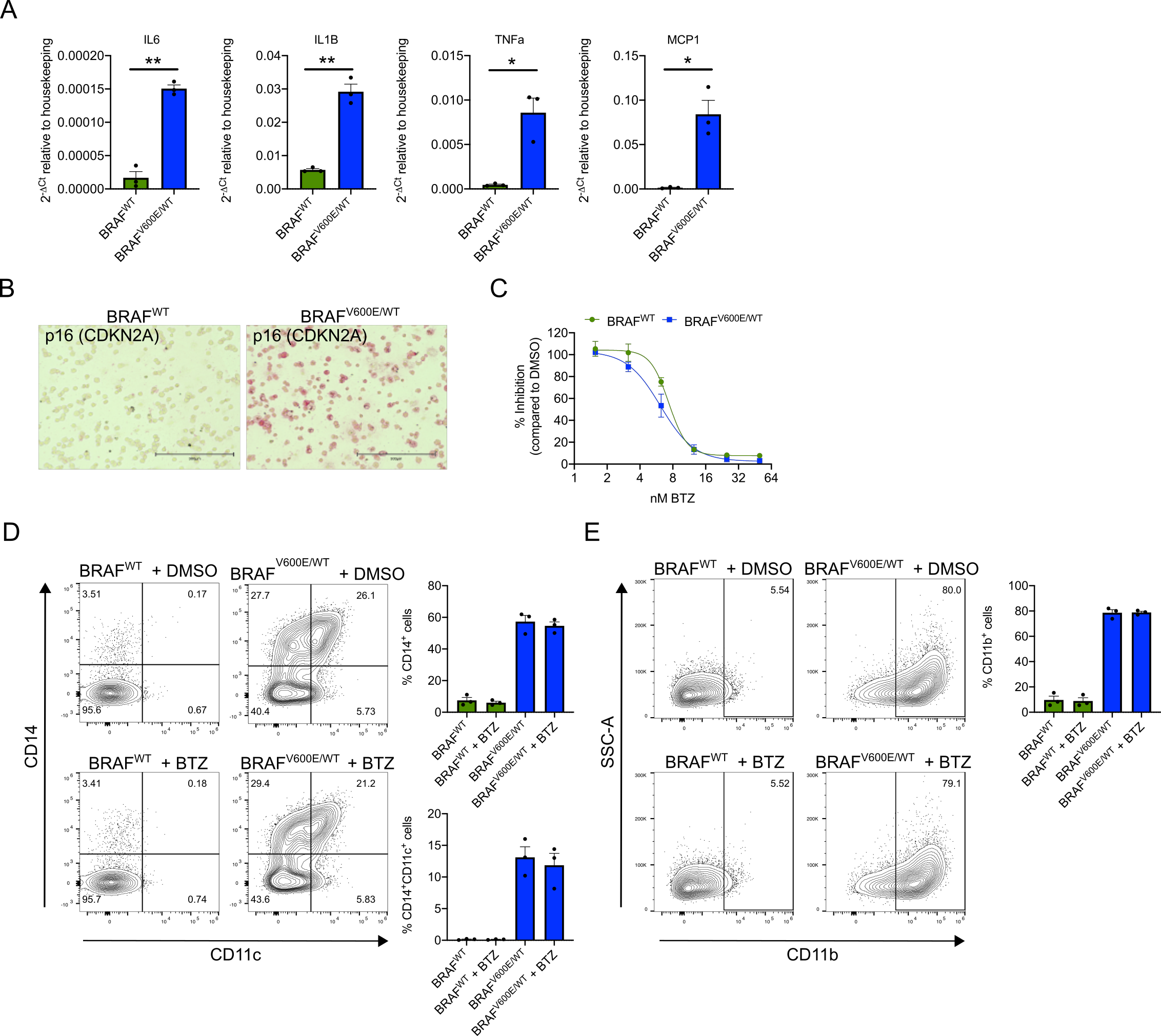
**

**Supplementary Figure 8. Bortezomib treatment of *BRAF^WT^* and *BRAF^V600E/WT^* HSPCs.** (A) Graphs depicting the relative expression of SASP cytokines (n=3). *GAPDH* was used as housekeeping gene. (B) Representative images depicting the expression of p16 (CDKN2A) in *BRAF^V600E/WT^* and *BRAF^WT^* cells cultured for 6 days in HSPC retention medium. (C) Graph depicting a dose-response of sort-purified *BRAF^WT^* and *BRAF^V600E/WT^* cells expanded for 8 days in HSPC retention medium and treated with BTZ for 48 hours compared to DMSO controls. Viability was measured with CellTiter-Glo^®^ (n=4). (D) Representative flow cytometry plots and graphs depicting the percentage of cells expressing CD14, CD11c, (E) and CD11b in cells cultured under HSPC retention medium in the presence or absence of 5 nM BTZ for 4 days (n=3). DMSO was used as a vehicle control in (C), (D), and (E). Data are shown as mean ± SEM. *P<0.05, **P<0.01.

**Supplementary Materials and Methods**

**Human CD34^+^ HSPC cell isolation and culture**

Human CD34^+^ HSPCs were isolated from umbilical cord blood (UCB) following mononuclear peripheral cell isolation by density gradient separation and magnetic enrichment (Miltenyi Biotech, Bergisch Gladbach, Germany) according to the manufacturer’s instructions. UCB was collected from donors during healthy, full term pregnancies. Informed consent was obtained and the ethical approval (IRB approval: 31-322 ex 18/19) was obtained from the Medical University of Graz.

HSPCs were cultured in StemSpan SFEMII (StemCell Technologies, Vancouver, Canada) supplemented with 0.2% penicillin/streptomycin (P/S), 100 ng/mL of human recombinant stem cell factor (SCF), thrombopoietin (TPO), FMS- like tyrosine kinase 3 ligand (FLT3L), and interleukin 6 (IL-6), 750 nM StemReginin 1 (SR1; Peprotech, Rocky Hill, NJ, USA) and 35 nM UM171 (StemCell Technologies). Supplemented SFEMII medium will be referred to as HSPC retention medium.

**Single guide RNA (sgRNA) design**

The sgRNA was designed to target the intron 14 of the *BRAF* gene. Target sequence: 5’- AGACCTCTGACCTTGCTCAG-3’. The synthetic chemically modified sgRNA was acquired from Synthego (Redwood City, CA, USA).

**Recombinant adeno-associated virus serotype 6 (rAAV6) production**

HDR templates containing either the complementary DNA (cDNA) BRAF^V600E^ or BRAF^WT^ spanning from exon 15 to exon 18 were cloned into the pAAV-MCS plasmid (Agilent #240071, Santa Clara, CA, USA). Upstream of the cDNA a splice acceptor sequence including the 3’ splice-site, branch point and the polypyrimidine tract of intron 14 was added. Downstream of the cDNA a fluorescent reporter protein (GFP or BFP) under the control of the SFFV promoter was added. The HDR template was flanked by left and right homology arms matching the Cas9 target site. rAAV6 was produced as previously described[1] by transfecting HEK293T cells with pDGM6 and the pAAV-MCS plasmid containing the HDR template and the virus was extracted 48 hours later by using the AAVpro Purification Kit (Takara Bio Inc, Shiga, Japan) according to the manufacturer’s instructions.

**CRISPR/Cas9 gene editing and rAAV6 transduction**

Following a 72-hour expansion in HSPC retention medium, 0.5x10^6^–3x10^6^ cells were suspended in 100 μL of human primary P3 nucleofector solution (Lonza, Basel, Switzerland) containing 15 μg Cas9 (IDT, Coralville, IA, USA) pre-complexed with BRAF sgRNA (1:2.5 molar ratio) and electroporated using the program DZ-100 of the Lonza 4D Nucleofection system. The cells were transduced by adding the rAAV6 virus directly to the cells in culture at a concentration of 5,000-10,000 vector genome copies/cell. Following 6-8 hours of incubation, the medium was replaced with fresh HSPC retention medium. Cells expressing high levels of the reporter fluorescent proteins were FACS-sorted after 48 hours.

**Genomic DNA extraction**

Genomic DNA was extracted by suspending 2x10^5^ cells in 50 μL of Quick Extract DNA Extraction Solution (Lucigen). Cells were first vortexed and incubated for 6 minutes at 65°C followed by a second vortexing step and subsequently incubated for 2 minutes at 98°C.

**Immunoblotting**

Cells were lysed by incubating the cells in RIPA lysis buffer supplemented with protease and phosphatase inhibitor cocktail. Following a 30 minute incubation on ice, the lysates were centrifuged for at 4°C at 15,000 r.p.m. Supernatants were collected and protein concentration was determined using the Lowry method. Proteins were separated on a mini-Protean TGX precast 4-20% gel (Bio-Rad, Hercules, CA, USA) and the Bio-Rad Trans Blot TurboBlotting system was used for transfer to a membrane. Membranes were first blocked for 1 hour in 5% BSA and then incubated overnight with primary pERK1/2, ERK1/2 (Cell Signaling Technology, Danvers, MA, USA), and with GAPDH at room temperature for 1 hour. Subsequently, membranes were incubated with either an anti-mouse or anti-rabbit IgG HRP-linked secondary antibody (Cell Signaling Technology) at RT for 1 hour. Detection was performed with the Clarity Western ECL Substrate (Bio-Rad) and images were acquired with the ChemiDoc imaging system (Bio-Rad).

**RNA sequencing**

Total RNA was extracted by using the Monarch Total RNA Miniprep Kit (NEB, Ipswich, MA, USA) according to the manufacturer’s instructions following a 48 hour culture of 1.5x10^5^ sort-purified modified HSPCs in HSPC retention medium. RNA-seq libraries were prepared with the TruSeq Stranded mRNA LT sample preparation kit and sequenced on a HiSeq 3000 instrument (Illumina, San Diego, CA, USA). NGS reads were mapped using STAR and differential gene expression was calculated with DESeq2. Gene set enrichment analysis was performed by using GSEA (v.4.1.0). Raw sequencing data were deposited in NCBI's Gene Expression Omnibus (GEO; GSE230040).

**Colony forming unit (CFU) assay**

Sorted, modified HSPCs were suspended in methylcellulose medium (MethoCult™ M3434, StemCell Technologies) at a concentration of 300 cells/mL and incubated at 37°C/5%CO_2_ for 14 days. Experiments were performed in triplicates. Colonies were counted and scored either manually or by using the STEMvision instrument (StemCell Technologies).

**Myeloid differentiation assay**

Sorted, modified CD34^+^ HSPCs were cultured for 5 days in STEMSpan SFEMII supplemented with 1% P/S, 100 ng/mL SCF, 100 ng/mL FLT3L, 100 ng/mL TPO, 20 ng/mL IL-6, 20 ng/mL IL-3, 20 ng/mL granulocyte-macrophage colony-stimulating (GM-CSF), 20 ng/mL G-CSF, and 1 μM Hydrocortisone.

**LC differentiation**

For CD34^+^ stem cell-derived LC differentiation assays, cells were differentiated under serum-free conditions as previously described[2, 3]. For LC differentiation assays in the presence of the Notch agonist Jagged-2 (Jag2; R&D Systems, Minneapolis, MN, USA), cells were first expanded for 6 days in HSPC retention medium and then 4x10^4^ cells were cultured for 6 days in Jag2 coated plates in RPMI-1640 (Sigma Aldrich, St. Louis, MO, USA) supplemented with 10% fetal bovine serum (FBS; PAN Biotech, Aidenbach, Germany), 1% P/S, 100 ng/mL GM-CSF (Peprotech), and 1 ng/mL transforming growth factor-1 beta (TGF-β1; R&D Systems). The plates were coated as previously described[4, 5].

**Xenotransplantation**

5x10^5^ HSPCs engineered with *BRAF^WT^* or *BRAF^V600E^* were intrafemorally transplanted into sub-lethally irradiated immune-compromised NOD/SCID IL2rg-/- KitW41/W41 (NSGW41) mice. Mice were analyzed for human hematopoietic cell engraftment by flow cytometric analysis of bone marrow aspirates on week 8. If not otherwise stated, mice were sacrificed on week 20 for organ collection and histological analysis of spleen and liver was performed. All animal experiments were performed in accordance with the EU Directive 2010/63/EU and are approved by the Austrian Federal Ministry of Science, Research, and Economy (Vienna, Austria, BMWFW-66.010/018-V/3b/2018).

**Flow cytometry**

Cells were collected, washed with 1X DPBS, and suspended in 100 μL of staining buffer (DPBS + 0.1% BSA). Fc receptors were blocked by incubating the cells for 5 minutes on ice with 5 μL of human FcR blocking reagent (BD Biosciences, San Jose, CA, USA). Surface markers were stained with fluorochrome-labeled antibodies and incubated on ice for 20 minutes. Data were acquired with the CytoFLEX S or CytoFLEX LX cytometers (Beckman Coulter, Brea, CA, USA) and analyzed with the FlowJo™ software (BD Biosciences). A full list of antibodies used for flow cytometry can be found in **Supplementary Table 1**. Aria IIIu cell sorter (BD Biosciences) was used for sort-purification.

**Immunohistochemistry**

Spleen and liver samples were fixed with 4% (w/v) neutral buffered formalin and incubated for 24h. Afterwards, they were embedded in paraffin (Tissue Tek Tec, Sakura), sectioned (4 μM), deparaffinized, and stained with hematoxylin–eosin according to standard histopathological techniques[6].

Cytospin preparations were fixed with 4% (w/v) neutral buffered formalin for 10 min.

Immunohistochemistry was performed on deparaffinized tissue sections or formalin fixed cytospin preparations using a Dako Omnis (Dako, Hamburg, Germany) autostainer.

Anti-BRAF (mutated V600E) antibody [VE1] (Abcam ab228461) (Abcam, Cambridge UK): demasking EnV FLEX TRS, High pH at 95 °C for 24 minutes, primary antibody binding for 25 minutes, EnV FLEX Peroxidase-Blocking Reagent 3 minutes, detection using EnV FLEX+ mouse detection system and counterstained with hematoxylin. Anti-CD68 (DAKO Clone KP1, GA609, ready to use, Dako,): demasking EnV FLEX TRS, High pH at 95 °C for 24 min, primary antibody binding for 20 minutes, EnV FLEX Peroxidase-Blocking Reagent 3 minutes, detection using EnV FLEX+ mouse detection system and counterstained with hematoxylin. Anti-S100 (DAKO Ready-to-Use GA504, Dako): demasking EnV FLEX TRS, High pH at 95 °C for 24 minutes, primary antibody binding for 20 minutes, EnV FLEX Peroxidase-Blocking Reagent 3 minutes, detection using EnV FLEX+ mouse detection system and counterstained with hematoxylin .The Ventana Benchmark Ultra (Roche, Mannheim, Germany) autostainer was used for CD163 (Cell Marque, MRQ-26, Cell Marque, Rocklin, CA, USA) mouse monoclonal antibody incubation using the following conditions: demasking Ultra CC1 for 64 minutes, primary antibody binding for 32 minutes, detection using the UltraView system and counterstained with hematoxylin.

**Immunofluorescence**

Immunofluorescence staining was performed on 3.5 μm formalin-fixed paraffin embedded samples. Sections were deparaffinized and antigen retrieval was performed with Dako target retrieval solution. Samples were first blocked with Dako protein block serum-free (Agilent, Inc, Santa Clara, CA, USA) followed by a second blocking step using the ReadyProbes™ mouse on mouse IgG blocking solution (ThermoFisher) before incubation with 1:100 diluted rat anti-human CD207-AF488 antibody (Clone 929F3.01; Novusbio) overnight at 4°C. 4’-6-Diamidino-2-phenylindole (DAPI) was utilized as nuclei counterstain and images were obtained on a Nikon A1 confocal microscope.

**Real-time PCR (qPCR)**

Total RNA was reverse-transcribed into cDNA by using the LunaScript RT SuperMix Kit (NEB, Ipswich, MA, USA) according to the manufacturer’s instructions. Gene expression was analyzed by performing qPCR using the Luna Universal qPCR Master Mix in a C1000 Touch Thermal Cycler (Bio-Rad). Five ng of template cDNA was used and GAPDH was used as housekeeping gene. A full list of the primer sequences can be found in **Supplementary Table 2**.

**Dose-response curve**

Three thousand *BRAF^WT^* and *BRAF^V600E/WT^* cells were plated in an opaque-walled 96-well plate in 50 µL of HSPC retention medium. Fifty µL of HSPC retention medium containing BTZ or DMSO control at the desired concentration was added to each well and the cells were incubated at 37°C/5%CO_2_. Following a 48 hour incubation, 100 µL of CellTiterGlo^®^ was added to each well, mixed for 2 minutes on an orbital shaker, and incubated for 10 minutes at RT before measuring the luminescent signal.

**NF-κB reporter studies**

U937 NF-κB reporter cells expressing a 5 × NF-κB–GFP reporter cassette were obtained as previously described[7].

**Statistical analysis**

GraphPad 6 (GraphPad Software Inc, San Diego, CA, USA) was used to statistically analyze data. Comparisons between two groups were analyzed by Two-tailed Student’s t-test. Comparisons between more than two groups were analyzed by one-way ANOVA followed by Sidak’s multiple comparison test or two-way ANOVA for multiple comparisons with different time-points . P values below 0.05 were considered to be statistically significant.

**Data sharing statement**

RNAseq data were deposited in NCBI's Gene Expression Omnibus (GEO; GSE230040). Original data are available from the corresponding author upon reasonable request.

**References**

1. Sconocchia T, Foßelteder J, Köhnke T, Majeti R, Reinisch A. Engineering Oncogenic Heterozygous Gain-of-Function Mutations in Human Hematopoietic Stem and Progenitor Cells. J Vis Exp. 2023;e64558.

2. Sconocchia T, Hochgerner M, Schwarzenberger E, Tam-Amersdorfer C, Borek I, Benezeder T, et al. Bone morphogenetic protein signaling regulates skin inflammation via modulating dendritic cell function. J Allergy Clin Immunol. 2021;147:1810-1822.e9.

3. Sconocchia T, Del Gaudio I, Meshcheryakova A, Mechtcheriakova D, Wadsack C, Strobl H. Induction of the sphingosine-1-phosphate signaling pathway by TGF-β1 during Langerhans-type dendritic cell differentiation. Eur J Immunol. 2021;51:1854–1856.

4. Varnum-Finney B, Wu L, Yu M, Brashem-Stein C, Flowers D, Griffin JD, et al. Immobilization of Notch ligand, Delta-1, is required for induction of notch signaling. J Cell Sci. 2000;113:4313–4318.

5. Schwentner R, Jug G, Kauer MO, Schnöller T, Waidhofer-Söllner P, Holter W,et al. JAG2 signaling induces differentiation of CD14 + monocytes into Langerhans cell histiocytosis-like cells. J Leukoc Biol. 2019;105:101–111.

6. Cook CH. Origins of ... tinctorial methods in histology. J Clin Pathol. 1997;50:716–20.

7. Jörgl A, Platzer B, Taschner S, Heinz LX, Höcher B, Reisner PM, et al. Human Langerhans-cell activation triggered in vitro by conditionally expressed MKK6 is counterregulated by the downstream effector RelB. Blood. 2007;109:185–193.

**Supplementary Tables**

**Supplementary table 1. Flow cytometry antibodies**

| **Marker** | **Clone** | **Fluorophore** | **Reactivity** | **Company** |
| --- | --- | --- | --- | --- |
| CD14 | RM052 | APC/PC7/ECD | Human | Beckman Coulter |
| CD11b | Bear1 | PC7 | Human | Beckman Coulter |
| CD88 | S5I1 | PE | Human | Biolegend |
| CD1c | L161 | APC | Human | Biolegend |
| CD16 | 3G8 | PE-Cy5 | Human | BD  Bioscences |
| CD11c | B-ly6 | PE | Human | BD Biosciences |
| CD1a | HI149 | APC | Human | BD Biosciences |
| CD207 | MB22-9F5 | PE | Human | Miltenyi  Biotec |
| CD34 | 581 | PC7 | Human | Beckman Coulter |
| CD45 | HI30 | BB700 | Human | BD Biosciences |
| CD33 | WM53 | PE | Human | BD Biosciences |
| CD19 | SJ25C1 | SB600 | Human | Invitrogen |
| CD45 | 30-F11 | APC-Cy7 | Mouse | BD Biosciences |
| Ter119 | Ter-119 | BUV661 | Mouse | BD Biosciences |
| 7-AAD | - | - | - | BD Biosciences |
| SYTOX™ Red | - | - | - | Thermo  Fisher |

**Supplementary table 2. Primer list real-time PCR**

| **Gene** | **Forward primer** | **Reverse primer** |
| --- | --- | --- |
| IL6 | CCACACAGACAGCCACTCAC | TGCCTCTTTGCTGCTTTCAC |
| TNF | ACGCTCTTCTGCCTGCTG | CTTGTCACTCGGGGTTCG |
| MCP1 | CTGTGATCTTCAAGACCATTGTG | AGTTTGGGTTTGCTTGTCCAG |
| IL1B | TGGCAATGAGGATGACTTGTTC | TGGTGGTCGGAGATTCGTAG |
| GAPDH | GTCTCCTCTGACTTCAACAGCG | ACCACCCTGTTGCTGTAGCCAA |
